# Maternal Nutrition During Late Gestation and Lactation: Association With Immunity and the Inflammatory Response in the Offspring

**DOI:** 10.3389/fimmu.2021.758525

**Published:** 2022-01-21

**Authors:** Qihui Li, Siwang Yang, Xiaoli Zhang, Xinghong Liu, Zhihui Wu, Yingao Qi, Wutai Guan, Man Ren, Shihai Zhang

**Affiliations:** ^1^ Guangdong Provincial Key Laboratory of Animal Nutrition Control, College of Animal Science, South China Agricultural University, Guangzhou, China; ^2^ College of Animal Science and National Engineering Research Center for Breeding Swine Industry, South China Agricultural University, Guangzhou, China; ^3^ Guangdong Laboratory for Lingnan Modern Agriculture, South China Agricultural University, Guangzhou, China; ^4^ College of Animal Science, Anhui Science and Technology University, Anhui Provincial Key Laboratory of Animal Nutritional Regulation and Health, Fengyang, China

**Keywords:** maternal nutrition, neonate, growth, disease resistance, inflammatory, immunoglobulin

## Abstract

The immature immune system at birth and environmental stress increase the risk of infection in nursing pigs. Severe infection subsequently induces intestinal and respiratory diseases and even cause death of pigs. The nutritional and physiological conditions of sows directly affect the growth, development and disease resistance of the fetus and newborn. Many studies have shown that providing sows with nutrients such as functional oligosaccharides, oils, antioxidants, and trace elements could regulate immunity and the inflammatory response of piglets. Here, we reviewed the positive effects of certain nutrients on milk quality, immunoglobulin inflammatory response, oxidative stress, and intestinal microflora of sows, and further discuss the effects of these nutrients on immunity and the inflammatory response in the offspring.

## Introduction

During gestation and lactation, maternal nutrition is a predominant factor to regulate the growth and immunity of piglets (1, 2). Since neonates are born without brown fat reserves, timely intake of colostrum is the guarantee of energy supply for piglets. In addition, colostrum also provides bioactive molecules such as immunoglobulins and inflammatory factors to piglets (3). Even though maternal immunoglobulins cannot cross the placental barrier (4), these immunoglobulins could transfer to piglets through colostrum and milk (5). Maternal diets regulate the composition of colostrum and milk, which further affect the maturation of immune system in neonates (6). Furthermore, maternal milk-derived cytokines also regulate the immunity of neonates (6). It is worth noting that maternal intestinal microflora play a crucial role in regulation of immune development and response during the neonatal period (7). Transferring the intestinal flora of sows during pregnancy into sterile mice improved the intestinal innate immunity and reduced the inflammatory response in their offspring (8). Interestingly, newborn intestinal bacteria is derived from maternal microbiota during delivery and lactation (9). Thus, the regulation of maternal intestinal microflora by nutrients indirectly affect the offspring immunity and inflammatory response.

Maternal infection or inflammatory exposure during pregnancy impairs the innate response of newborns and increases their susceptibility to infection (10). During pregnancy, sows undergo dramatic changes of physiological metabolism and immunity (11),with markedly increased oxidative stress and inflammatory response (12). Imbalanced inflammatory response are closely related to reproductive disorders, including constipation, abortion and intrauterine growth retardation (9). In addition, inflammatory factors could transfer from maternal to fetus and regulate immunity and inflammatory response. Thus, modification of dietary components of sows during pregnancy might affect neonate intestinal development, immunity, and inflammation. In this review, we summarized the recently published data regarding prebiotic and nutrient supplementation to sow diets during late gestation (mainly during G85-G114) and lactation on maternal milk quality, inflammatory response, oxidative stress and then discuss their effect on the inflammatory response and immunity in the offspring.

## Soluble Dietary Fiber

**Figure 1:**
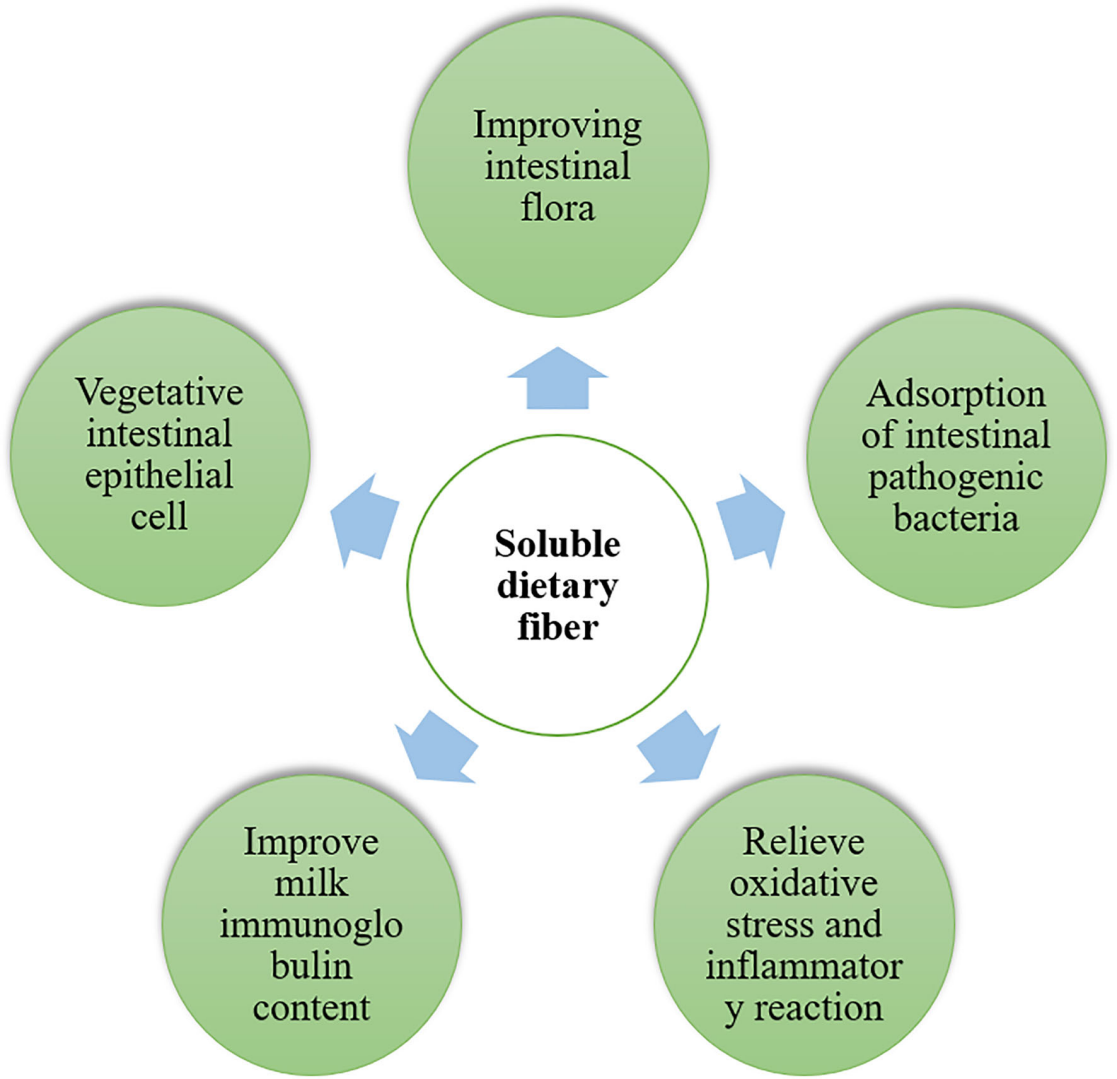
The soluble dietary fibers beneficial to intestinal health of sow, improves colostrum quality, enhance antioxidant capacity of sows and reduces inflammatory reaction of piglets.

As indigestible carbohydrate, dietary fiber (DF) is partially or completely fermented by microorganisms in the large intestine, which could be categorized into insoluble and soluble fiber (13). Insoluble fiber speeds up the intestinal circulation, reduces constipation and increases the intestinal volume (14). While soluble fiber is fermented to produce numerous functional metabolites, such as short-chain fatty acids (SCFAs), which could be transmitted from maternal to offspring. Among them, acetate regulates intestinal permeability and anti-inflammatory effect (15). Butyrate improves intestinal morphology, promotes beneficial bacterial growth, and enhances immune defense (16, 17). It has also been shown that maternal DF supplementation could promote T cell differentiation and reduce the inflammatory response in the offspring by regulating the intestinal microbial composition (18). In this review, we focused on the effects of several representative DF supplementation in sow diets (**Table 1** and **Figure 1**).

**Table 1:** Maternal microbial and soluble dietary fiber intake in the regulation of neonatal infection, immunity and production performance.

Breed, feeding time and products	Reproductive and lactation performance	Immune and oxidative stability of sows and piglets	Intestinal health and inflammation	Others	References
**Breed:** Large White × Landrace **period:** G85-G110 **Product:**isomaltooligosaccharide 5.0 g/ kg IMO 0.2 g /kg B. subtilis 0.2 g/ kg B. licheniformis	**Reproductive performance** ↑weaning BW (45.63-55.18 kg) Average litter gain (28.43-35.87 kg) **Milk** ↑Total milk yield (113.73-143.46 kg) IgM (1 794.18-1 894.73 g/ mL) on L0 IgA (607.50-922.07 g /mL) on L17	**N/A**	**N/A**	**Sow plasma (L17)** ↓ALT (37.23-35.49 U/ L) ALP (40.23-31.82 U /L)	(19)
**Breed:** Yorkshire **period:** G85-L21 **Product:**chitosan oligosaccharide (100 mg/kg COS)	**Reproductive performance** ↑daily BW gain: (1.90-2.21 kg) piglet weaning weight: (53.63-60.04 kg) **Colostrum (L1)** ↑Solids-not-fat: (128.07 -153.33 g/kg) IgM: (3.27-4.76 g/L) **Milk (L21)** ↑Lactose: (44.12 -56.10 g/kg) Solids-not-fat: (85.44-101.82 g/kg)	**Sow serum (L1)** ↑CAT: (14.25-20.49 U/mL) T-AOC: (5.93 -8.79 U/mL) IL-10: (50.57-67.73 pg/mL) IgA: (71.31-91.48 μg/mL) IgM: (92.53-117.86 μg/mL) ↓MDA: (16.97-11.90 nm/mL)	**N/A**	**N/A**	(20)
**Breed:** Yorkshire × Landrace **Period:** G85-L21 (weaning) **Product:** Sugar beet pulp (SBP) 20% SBP in gestation and 10% SBP in lactation	**Piglet at weaning (L21)** ↑Litter weight: (56.94-64.39 kg) Weaning weight: ((5.74-6.26 kg) ADG: (196-221 g/d) **Colostrum (L1) ** ↑IgA: (7.94-9.17 g/L)	**Piglet serum (L21)** ↓DAO: (5.68-3.60 U/L) Endotoxin: (0.60-0.47 EU/ml) IL-6: (178.49-154.30 pg/mL) TNF-α: (102.45-80.28 pg/mL) ↑IL-10: (4.55-5.13 pg/mL) **Piglet ileum (L21)** ↓TNF-α: (1-0.6) IL-6: (1-0.6) ↑IL-10: (1-1.3) SIgA: (0.8-1.5 μg/mg)	**Piglets Ileal Tight Junction (L21) mRNA expression** ↑Occluding: (1-1.3) ZO-1: (1-1.4) **Piglet Jejunum** ↑Villus height: (387-447 μm) **Intestinal microbiota (Piglet)** the relative abundance of Christensenellaceae was increased significantly	↑Sow ADFI: (4.80-5.48 kg/d) **Piglet serum (L21)** ↑GH: (3.37-4.23) IGF-1(156.09-187.86)	(21)
**Breed:** Yorkshire × Landrace **Period:** G83-L28 (weaning) **Product:** seaweed-derived polysaccharides (10·0 g SDP/d)	↑gestation period: (113.5-114.5 d)	**Piglet ileum (weaning) gene expression** ↓PEPT1: (1.71-0.43) GLUT1: (0.98-0.65) GLUT2: (1.76-0.41) ↑IL-1: (0.99-2.85) IL-12A (p35): (0.97-1.93) TNF-α: (0.93-1.66) ↓IL-10: (0.82-0.29) IL-6: (1.12-0.64) IL-8: (1.15-0.77)	**Log GCN/g of sow faece** ↓Enterobacteriaceae: (8.55-7.76) parturition **Piglet weaning** ↑villus height: (347-466 μm) ileum villus height: (317-454 μm) jejunum ↓crypt depth: (144-108) ileum	Piglets had a lower diarrhoea score during the lactation period	(22)
**Breed:** Large White x Landrace **Period:** G107-L26 **Product:** seaweed extract (10 g/d)	**Colostrum ** ↑IgA: (8.02-11.61 mg/mL)	**Piglet serum (L14)** ↑IgG: (8.59-11.36 mg/ml)	**piglet intestinal microbiology (weaning)** ↓colonie E.col: (6.45-5.11 Log cfu/g) ↑lactobacilli: E.coli: (1.21-1.45 Log cfu/g)	**log cfu/g of sow feces (farrowing)** ↓En-terobacteriacea: (8.60-7.26)	(23)
**Breed:** Landrace sows **Period:** G85-L21 (weaning) **Product:** 2.0% pregelatinized waxy maize starch plus guar gum (SF)	**Piglet at weaning (L21)** ↑Final BW: (6.49-7.09 kg) ADG: (233.66-261.20 g/day)	**Piglet serum (L14)** ↓IL-6: (310-290 pg/ml) ↑TGF-β: (650-750 pg/ml) IL-10: (110-140 pg/ml)	**Piglet plasma (L14)** ↓Zonulin: (700-550 ng/ml) Endotoxin: (0.7-0.5 Eu/ml) Diamine oxidase: (10-9 U/L) ↓**lipocalin-2 (80-58μg/g feces)** **Intestinal microbiota (Piglet)** strong increase in relative abundance of the Lactobacillus genus	**Piglet Diarrhea rate:** (13.69-10.35%) **plasma hormone of piglets (L14)** ↑GH: (587.65-657.49 pg/ml) IGF-1: (309.04-374.63 ng/ml)	(24)
**Breed:** Large White × Yorkshire **Period:** Sow: G86-L20 Piglet: D7-D35 **Product** Mannan oligosaccharide Sow: 400 mg/kg Piglet: 800 mg/kg	**N/A**	**Piglet serum (D35)** ↓IL-2: (146.58-107.83 ng/L) IL-4: (18.21-12.09 ng/L) IFN-γ: (535.58-448.88 ng/L) ↑IL-10: (65.82-76.04 ng/L)	**Intestinal microbiota (Piglet on D35)** log10 counts of Lactobacillus, E. coli **↓**E. coli: (6.83-6.43 Jejunum) ↑Lactobacillus:(7.63-8.44 in Jejunum) (7.82-8.76 in Cecum) **immunoglobulin A in piglet jejunum** ↑sIgA: (4.48-6.77 mg/g pro)	**N/A**	(25)
**Breed:** Landrace×Yorkshire **Period:** G86-L21 **Product:** chitosan oligosacchari (30 mg/kg)	**Colostrum (L1)** IgM: (0.95-1.3 g/L) **Umbilical cord blood** IgM: (38.36-43.26 g/L)	**Piglet serum (D21)** ↑IL-10: (57.04-65.29 ng/L) IgG: (163.81-192.29 mg/L) C3: (211.35-254.35 mg/L)	**N/A**	**N/A**	(26)

↑, increase; ↓, decrease; N/A, No Value; BW, body weight; IgA, Immunoglobulin A; IgG, Immunoglobulin G; IgM, Immunoglobulin M; T- AOC, Total antioxidant capacity; CAT, Catalase; MDA, Malondialdehyde; IL-10, interleukin 10; IL-6, interleukin 6; IL-8, interleukin 8; IL-4, interleukin 4; IL-2, interleukin 2; TNF-α, tumor necrosis factor-α; GH, growth hormone; IGF-1, insulin like growth factor 1; ZO-1, zonula occludens-1; ALT, cereal third transaminase; ALP, alkaline phosphatase; ADG, average daily gain; PEPT1, peptide-transporters 1; GLUT1, glucose transporter-1; GLUT2, glucose transporter-2; TGF-β, transforming growth factor β; IFN-γ, interferon-γ; C3, complement 3; sIgA, secretedimmunoglobulin A.

Isomaltooligosaccharide (IMO) has been reported to activate the immune system (27) and promotes the proliferative potential of beneficial bacteria (particularly *Bifidobacterium*) of sows (28). A recent study reported that feeding sow IMO during late pregnancy (G85-G110) could promote milk GH, IgA and IgG concentrations, increase litter average daily gain (ADG) of piglets, and reduce backfat loss in sow during lactation (19). Similarly, another study showed that IMO given to sows during late pregnancy increased the concentration of IgA, IgG and IgM in colostrum and reduced the diarrhea rate of piglets (29).

Chitosan oligosaccharide (COS) has good water solubility and performs antioxidant (30), anti-inflammatory (31), and immunity-enhancing functions (32). During gestation and lactation (G85-L21), sows given to COS (100 mg/kg) have higher milk production as well as IgM and lactose concentration in colostrum. In addition, COS (100 mg/kg) increased total number of piglets born and weaning weight per litter (20). Importantly, feeding sows with 30 mg/kg or 100 mg/kg COS both increase the serum IgG concentration of piglets, which indicates the enhancement of immune function in neonates (26, 33).

Sugar peat pulp (SBP) contains large amounts of soluble fibers such as pectin and dextran (34). Feeding SBP could increase the feed intake of sows during lactation by improving insulin sensitivity, which is beneficial to the serum GH and IGF-1 levels and growth of piglets (35). SBP supplementation (20% during gestation and 10% during lactation) reduced pro-inflammatory cytokines (IL-6 and TNF-α) in serum of sow. Consistently, pro-inflammatory cytokines (IL-6 and TNF-α) in colostrum, milk and piglet serum are also decreased. Moreover, SBP supplementation in sow diet increase intestinal SCFA and colostral IgA levels, which might be beneficial for reducing inflammatory response in piglets (21).

Seaweed extracts (SWEs) mainly consists of seaweed polysaccharide (SDP), laminarin, and fucoidan (36). Supplementation with SWEs from late gestation to weaning increased colostrum IgG and IgA concentrations. Correspondingly, higher serum IgG concentrations were observed in piglets, which indicates the increased immune function (23). Sudden weaning of piglets is often accompanied by adverse morphological changes in the structure of the small intestine, including villous atrophy and crypt hyperplasia (37). Recent studies have shown the addition of seaweed-derived polysaccharides (10 g/d) to sow feed significantly increased the VH and ratio of villi/crypt (VH:CD) of weaned piglets. In addition, maternal SWE supplementation increases anti-inflammatory (TGF-β1) and inhibits pro-inflammatory factors (IL-6 and IL-8) in the ileum and colon of piglets. Accordingly, the diarrhea score of the piglets during lactation was decreased (22). Furthermore, SWEs diet reduced the number of *Enterobacteriaceae* in sow feces at delivery and the number of *Escherichia coli* in piglet feces at weaning (38). These benefits might be attributed to laminarin could agglutinate certain pathogens and inhibit their adhesion to mucosal epithelial surfaces (39).

Guar gum is a kind of galactomannan extracted from guar endosperm. It has high viscosity and water solubility, which is widely used as a stabilizer and thickener in foods (40). Feeding 2.0% guar gum diet to sow during the gestation and lactation period (G85-L21) could improve the intestinal barrier function, accelerate the growth and reduce the diarrhea rate of piglets. In addition, guar gum increases the abundance of *Lactobacilli* and decrease the abundance of *Bilophila spp* in intestine. Importantly, IL-10 and TGF-β levels were increased in piglets, which avoids over-activated immune system in piglets (24).

Mannan oligosaccharide (MOS), derived from the cell wall of Saccharomyces cerevisiae, has been used as a prebiotic for a long time (41). Recent supplementation of MOS in sow diets has been reported to regulate immunity and the inflammatory response in the offspring. Compared with the control treatment, MOS treatment (400 mg/kg) shortened the weaning estrous of the sows and increased the weaning weight of the piglets. Besides, sows fed MOS increased IgA, IgG, IgM in colostrum, and serum IgA and IgG levels in suckling piglets (25). Additionally, another study shows that the addition of MOS (400 mg/kg) to sow diet could significantly increase the sIgA content in jejunum mucosa and reduce the intestinal inflammatory response of piglets by inhibiting the TLR2/TLR4/NF-κB p65 pathway. Furthermore, MOS supplementation in sow diet increased the number of *Lactobacilli* and decreased the number of *Escherichia* coli in the jejunum of piglets, which is beneficial for reducing diarrhea (42).

Besides soluble fiber, insoluble dietary fiber also plays a crucial physiology role in sow. Insoluble dietary fiber accelerates gastrointestinal motility, reduces constipation and increases satiety of sows (43). Wheat bran (WB) is a insoluble fiber rich in arabinoxylan and cellulose, and widely used in the sow diet (44). A recent study showed that feeding WB to sows during late pregnancy and lactation (from G110 and L21) reduced inflammatory responses with the downregulation of serum IL-6 concentrations (21). In addition, the addition of wheat bran (25% during gestation and 14% during lactation) to sow diets increase the duodenal villi and higher colonic and ileal VH:CD ratios of the weaning piglets (45).

However, excessive level of dietary fiber could negatively affect total tract nutrient digestibility in pigs (46). As soluble fiber might increase digesta viscosity and slow down the diffusion of digestive enzymes in the small intestine (47). While insoluble fiber could promote the passage rate of chyme and reduce the mixing time of digestive enzymes and dietary ingredients (47). Therefore, overmuch high-fiber diet may cause reduced nutrient absorption by sows, which is detrimental to piglets. And the optimal dosage of fiber supplement in the diet of gestational sows needs further study.

## Oils

During late pregnancy and lactation period, sows require more nutrients and energy for fetal growth and milk synthesis. Oil supplementation in sow diets could prevent excessive mobilization of body reserves (48), shorten the estrous interval, improve milk quantity (49), and increase the survival rate and daily weight gain of weaned piglets (50). In addition, some specific types of fatty acids also participate in metabolic regulation and perform antibacterial and anti-inflammatory effects (51). In this section, we discussed the role of three wildly used oils (soybean oil, fish oil and olive oil) in sow diet.

Soybean oil is rich in linoleic acid. The addition of 2% soybean oil during pregnancy increased the content of protein and lipid-free solids in colostrum (**Table 2**). Furthermore, supplementation of soybean oil in the lactating diets of sows also resulted in higher concentrations of protein in maternal milk (54), which may be due to fatty acids stimulate the development of mammary duct and alveolar structure (55). In addition, maternal soybean oil supplementation also improved the intestinal morphology, digestive enzyme activities, serum growth factor concentrations and even intestinal immune function of piglets with the upregulation of immune-related genes (*TLR-4*, *TLR-9* and *MyD88*) in the ileum (52, 56).

**Table 2:** Maternal fats intake in the regulation of neonatal infection, immunity and production performance.

Breed, feeding time and products	Reproductive and lactation performance	immune and oxidative Stability of sows and piglets	Intestinal health	others	References
**Breed:** Landrace × Yorkshire **Period:** G0-L20 **Product:** 2% soybean oil	**Colostrum** **↑**No-fat solids: (15.53-22.90%) Protein: (5.85-8.79%)	**Piglet ileum (After farrowing) Gene Expression** **↑**TLR-4: (1.00-1.48) TLR-9: (1.00-1.40) MyD88: (1.00-1.22)	**Piglet Jejunum (After farrowing)** **↑**Villous height: (717-923 μm) Crypt depth: (76-88 μm) **Piglet Colon (After farrowing)** **↑**Crypt depth: (32-41 μm) ↓VCR: (6.53-4.40) (villous height to crypt depth ratio)	**Sow plasma (After farrowing)** Prolactin: (262.00-432.70 ng/mL)	(52)
**Breed:** Large White × Landrace **Period:** G109-weaning (L26) **Product:** fish oil and seaweed extract (100 g of FO/d, 10.0 g of SWE/d)	**Colostrum (SWE)** ↑IgG: (63.27-69.84 mg/ml) **Milk (L12) (SWE)** ↑CP: (5.17-5.39%) **Milk (L12) (FO)** ↑Total n-34: (1.73-4.62%) Ratio n-6:n-3: (9.75-3.80%)	**Piglet serum (L5)** ↑IgG (SWE): (19.31-22.9 mg/ml) IgA (SWE): (2.51-3.13 mg/ml) ↓IgA (FO): (3.12-2.52 mg/ml) **Piglet serum (L12)** ↑IgG (SWE): (9.98-12.04 mg/ml)	**N/A**	**Piglet serum (L26)** ↑Total n-6: (0.99-0.16%) Total n-3: (1.43-0.030%) Ratio n-6:n-3: (0.61-0.232%)	(23)
**Breed:** large white × landrace **Period:** G84-L21 **Product:** Fish Oil (2%) Or Olive Oil (2%)	**Litter Performance** ↑Piglet BW: (1.33-1.58 kg) OO ↑Piglet mortality: (7.2-12.3%) FO ↓Piglet mortality: (7.2-2.2%) OO **Colostrum** ↑Fat: (4.84-5.69%) OO MDA: (3.9-5.8 nmol/ml) FO IL-1β: (14-20 ng/L) FO **Milk (OO)** ** **↑Fat: (6.77-8.08%) L10 Fat: (5.86-7.99%) L21 ↓IL-1β: (20-10 ng/L) L10 IL-1β: (18-6 ng/L) L21 **Milk (FO)** ↑MDA: (3.9-8 nmol/ml) L10 MDA: (3.8-8 nmol/ml) L21	**Sow plasma (FO)** ↑MDA: (2-2.25 nmol/ml) L0 MDA: (2-3.5 nmol/ml) L10 MDA: (1.5-2 nmol/ml) L21 **Piglet serum (FO)** ↑MDA: (2.75-4 nmol/ml) L0 MDA: (3-4 nmol/ml) L21 GSH-Px: (275-300 U/ml) L0 **Piglet serum (OO)** ↓IL-1β: (12-10 ng/L) L21 TNF-α:(90-80 ng/L) L21	**N/A**	**N/A**	(53)

↑, increase; ↓, decrease. N/A, No Value; TLR-4, toll-like receptor 4; TLR-9, toll-like receptor 9; MγD88, myeloiddifferentiationfactor88 IgG, Immunoglobulin G; IgA, Immunoglobulin A; IL-10, interleukin 10; TNF-α, tumor necrosis factor-α; MDA, malondialdehyde; IL-1 β, interleukin-1 β; T- AOC, total antioxidant capacity; GSH-Px, glutathione peroxidase IL-6, interleukin 6.

**Figure 2:**
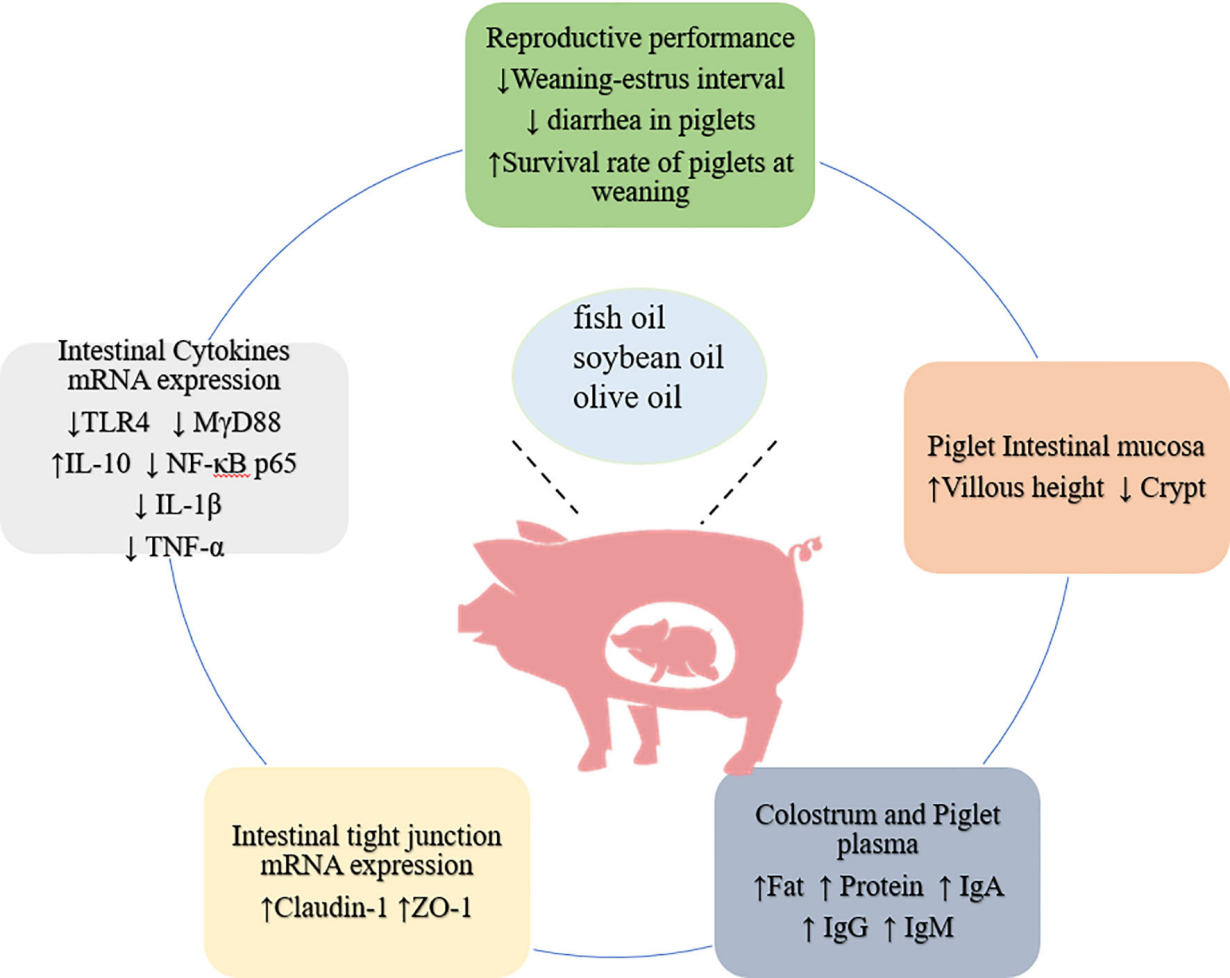
Beneficial effects of adding fat in feed of pregnant sow on piglets.

Fish oil (FO) is rich in long-chain n-3 polyunsaturated fatty acids, such as eicosapentaenoic acid (EPA) and docosahexaenoic acid (DHA), which have anti-inflammatory effects both *in vivo* and *in vitro* (57) (**Figure 2**). Maternal supplementation of FO accelerated immune system maturation and enhanced anti-inflammatory response of piglet (58). The addition of 3-5% fish oil to sow feed during lactation promoted the growth of piglets during lactation (59–61), which might partly due to the increased secretion of milk fat and immunoglobulins (IgM and IgG) (62, 63). Furthermore, fish oil also reduced the transmission of pro-inflammatory cytokines (IL-1β) from the sow to the piglets, and up-regulated the expression of IL-10 in the liver and pro-inflammatory cytokines (IL-6, TNF-α) in the skeletal muscle of piglets to alleviate the inflammatory response of the piglets (64, 65). However, addition of fish oil to sow diets could increase the sensitivity to oxidative stress in sows and piglets (66, 67). MDA is an indicator of lipid peroxidation, which is higher in the plasma of pregnant sows after feeding FO (53). This might due to unsaturated bonds in EPA and DHA were easily attacked by free radicals (68). Similar to fish oil supplementation, addition of n-3 PUFA during late pregnancy and lactation (G82-L22) reduced the weaning-estrous interval of sows, increased the concentrations of fat, protein and immunoglobulins (IgA, IgG and IgM) in milk (69). Furthermore, n-3 PUFA supplementation improved the intestinal barrier, reduced the diarrhea rate, and minimized the mortality of suckling piglets (69). Besides, changing the ratio of n-6/n-3 PUFA in the diet of lactating sows also affect the immune system and antioxidant status of piglets (70, 71).

Olive oil (OO) is rich in monounsaturated fatty acids (72), as well as antioxidant and anti-inflammatory components such as tocopherols, triterpenoid alcohols, phytosterols and phenolic compounds (73). Sows fed with olive oil (2% OO) diet during late pregnancy and lactation resulted in greater milk fat content, and higher birth weight and survival rate of piglets (53). This might be due to sows distributed a larger proportion of nutrients for fetus and neonate growth instead of using them for fat deposition. In addition, OO significantly reduced the contents of IL-1β, IL-6, MDA and TNF- α in milk, and improved the plasma levels of IL-1β and TNF-α in piglets (53). However, lower feed intake in sows was caused by OO feeding, which might be due to olive oil derived oleic acid upregulated plasma oleoyl ceramide (OEA) levels and caused anorexia in sows (74).

It is worth noting that high fat-induced obese sows have lower number of live-born piglets (75), piglet birth weight and weaning weight (76). Moreover, these piglets showed reduced responses to infection (77). One of the possible reasons is that obesity lead to lipotoxic placental environment (78, 79), which results in placenta proinflammatory response and oxidative stress (80, 81). The other reason is obese sow has higher plasma pro-inflammatory cytokines TNF-α, IL-1β, and IL-6 (75, 82). Maternal inflammation and oxidative stress further increase the expression of intestinal pro-inflammatory cytokines (83) and disrupts the homeostasis of immune cells (such as the number of T cells and macrophages) in the offspring (84), which makes them more vulnerable to inflammatory bowel disease. These data indicate that the excessive high-energy feed have catastrophic consequences for health of sows and piglets. Therefore, oil additive dosage should be considered in actual production.

## Antioxidants

During late pregnancy,rapid fetal development increases the metabolic burden and induces systemic oxidative stress of pregnant sows (85).Severe oxidative stress leads to postpartum hemorrhage, decreases neonate’s birth weight and even causes fetal death (86). Furthermore, oxidative stress usually causes inflammation and reduces immune system function in sows, which leads to growth-retarded fetuses (87, 88). The detrimental effect of maternal infection or inflammation on fetus development might be due to maternal inflammatory cytokines that transmitted from maternal to fetus (89, 90). Therefore, nutritional strategies to relieve oxidative stress in sows is crucial to improve fetus and neonate development (**Table 3**).

**Table 3:** Maternal antioxidant and other substrates intake in the regulation of neonatal Infection, immunity and production performance.

Breed, feeding time and products	Reproductive and lactation performance	immune and oxidative Stability of sows and piglets	Intestinal health	others	References
**Breed:** Large White × Landrace **period:** G80-L21 **Product:** grape seed polyphenols (300 mg/kg GSP)	**Reproductive performance** ↓dead fetuses: (1.19-0.63) ↑Farrowing survival: (81.47-89.32%) Preweaning survivability: (91.85-95.23%) **Colostrum** ↑IgM: (2.5-6 g/L) IgG: (38-80 g/L)	**Sow plasma (G110)** ↑SOD: (37.51-66.21 IU/mL) GSH-Px: (417.83-620.33 IU/mL)	**N/A**	**Sow plasma (G110)** ↑P4: (35-45 ng/ml) E2: (40-50 pg/ml)	(91)
**Breed:** Landrace × Yorkshire **period:** G85-L21 **Product:** fully oxidised β-carotene (8 mg/kg)	**Milk (14)** ↑Lactose: (5.67-6.09%) IgM: (0.024-0.057 g/L) **Colostrum** ↑IgM: (2.55-4.52 g/L) IgG: (29.91-33.22 g/L) IgA: (2.39-5.11 g/L) ↓TNF-α: (0.34-0.08 ng/mL) IL-8: (1079.06-605.46 pg/ml)	**N/A**	**N/A**	**N/A**	(92)
**Breed:** Landrace × Yorkshire **period:** G90-L21 **Product:** Rare Earth Elements (200 mg REE mixture/kg)	**Reproductive performance** ↓Within-litter birth weight CV: (0.21-0.18%) ↑Weight at 21st day: (5.71-6.21 kg) Daily weight gain: (223.06-241.75 g/day)	**Sow plasma (farrowing)** ↑GSH-Px: (650-700 U/ml) CAT: (4.8-6.5 U/mL) ↓TNF-α: (200-130 pg/ml) **Piglet plasma (weaning)** ↑SOD: (120-130 U/mL) ↓TNF-α: (120-80 pg/ml)	**Fecal Microbiota (lactating sows)** ↑Firmicutes: (78.2-81.0%) Bacteroidetes: (13-19.1%) **Piglet Fecal Microbiota (weaning)** ↓Proteobacteria phylum: (14.8-6.7%)	**Piglet plasma (weaning)** ↑IGF-1: (180-210 ng/ml)	(93)
**Breed:** Large White × Landrace **period:** G107-L21 **Product:** vitamin E (250 IU/kg)	**Reproductive performance** ↑BW of weaned piglets:(4·89-5·67 kg) Piglet Day 0-21 ADG: (160-194 g/d) **Colostrum** ↑Fat: (44·35-53·80 g/kg) IgG: (52·78-63·45 g/l) IgA: (8·02-9·01 g/l) α-tocopherol: (18·51-26·97 μg/l) **Milk** ↑Fat: (67·01-79·13 g/kg) IgG: (0·89-0·96 g/l) IgA: (3·81-4·11 g/l) α-tocopherol: (4.16-7.97 μg/l)	**Piglet plasma (L21)** ↑IgG (0·44-0·49 g/l) IgA (0·33-0·36 g/l) T-AOC (6·82-7·65 IU/ml) CAT (7·38-8·78 U/ml)	**N/A**	**N/A**	(94)
**Breed:** Yorkshire × Landrace **period:** G75-L21 **Product:** Taurine (1%)	**Reproductive performance** ↑Average daily gain: (194.62-230.11 g) Weaning weight: (5.35-6.29 kg) **Milk** ↑T-AOC: (106.21-165.16 U/ml) on L1 T-AOC: (34.45-105.93 U/ml) on L10 GP-x: (103.75-174.03 U/ml) on L1 CAT: (0.69-0.74 U/ml) on L10 T-SOD: (23.71-29.48 U/ml) on L10	**Piglet plasma (L1)** ↑T-SOD: (35.53-104.92 U/ml) T-AOC: (23.45-41.22 U/ml) CAT: (0.34-0.38 U/ml)	**Piglet Villous height** ↑Duodenum: (249.10-503.08 µm) on L1 Ileum: (318.61-467.21 µm) on L21 Jejunum : (358.39-524.045 µm) on L7 **villus height-to-crypt depth ratio** ↑Duodenum: (1.47-2.81) on L1 Jejunum: (1.38-1.99) on L7	**N/A**	(95)
**Breed:** Yorkshire × Landrace **period:** G85-L21 **Product:** lysozyme (300 g/t)	↓Stillborn: (0.89-0.15) Diarrhea rate: (2.24-1.41%) **Colostrum** ↑IgA: (3.21-3.51 mg/mL) **Milk (L7)** ↑IgA: (1.84-2.11 mg/mL)	**Sow plasma (L1)** ↑IgM: (0.81-0.98 mg/mL) **Piglet plasma (L21)** ↑IL-10: (209.60-239.21 ng/L) IgA: (2.16-2.56 mg/mL) IgG: (2.25-2.65 mg/mL) IgM: (23.98-28.87 mg/mL)	**N/A**	**N/A**	(96)
**Period:** G43-weaning **Product:** wheat bran (25% of WB in gestation and 14% of WB in lactation.)	**N/A**	**Piglet Ileal mRNA expression** ↑PPARγ: (1-1.37) IL6: (0.61-1)	**Piglet Small Intestine** ↑villi height: (380-450 μm) duodenum villi/crypt: (1.4-2) duodenum villi/crypt: (1.4-1.6) jejunum ↓crypts depth: (250-200 μm) jejunum	**N/A**	(45)
**Breed:** Large White × Landrace **Period:** G85-L20 **Product:** Yeast-based nucleotide (4 g YN/kg diet)	**Piglet at Weaning (D20)** ↑litter size: (9-10) ADG: (190-200 g) Sow total milk yield: (130-150 kg)	**Gene expression of Intestinal cytokine (neonatal piglets)** **Ileal** ↑(IL)-17: (1-1.8) IL-8: (1-1.5) TNF-α: (1-1.8) **Jejunal** ↑(IL)-17: (1-1.8) IL-6: (1-2.5) IL-8: (1-1.7) IFN-γ: (1-1.6) TNF-α: (1-1.8) **Duodenal** ↓IL-6: (1-0.5) ↑IL-1β: (1-1.6)	**Ileum (neonatal piglets)** ↑average villus height: (550-600 μm) villus height-to-crypt depth (V:C): (5-6) sIgA: (5-6.5 μg/g) **Intestinal tight junction (neonatal piglets) mRNA expression** **Ileal** ↓ZO-1: (1-0.6) **Jejunal** ↓ZO-1: (1-0.7) claudin-1: (1-0.5) **Duodenal** ↓claudin-1: (1-0.5)	↓ Diarrhoea rate of piglets: (4.5-3%)	(97)

↑, increase; ↓, decrease. N/A, No Value; SOD, superoxide dismutase; GSH-Px, glutathione peroxidase; P4, progesterone; E2, estradiol; IgM, Immunoglobulin M; IgG, Immunoglobulin G; IgA, Immunoglobulin A; TNF-α, tumor necrosis factor-α; IL-8, interleukin 8; IL-6, interleukin 6; IL-10, interleukin 10; IL-17, interleukin 17; IFN-γ, interferon-γ; CAT, catalase; IGF-1, insulin like growth factor 1; T- AOC, total antioxidant capacity; T-SOD, total Superoxide dismutase; ZO-1, zonula occludens-1; IL-1 β, interleukin-1 β; ADG, average daily gain; sIgA, secretedimmunoglobulin A; PPARγ, peroxisome proliferator-activated receptor γ.

Vitamin E, one of the most effective antioxidants, could directly react with free radicals and stimulate the expression of antioxidant enzyme genes, like GSH-Px and CAT (94). In addition, vitamin E enhances cellular and humoral immune responses in a variety of animals, including pigs (98, 99). During last week of gestation and lactation, vitamin E (250 IU/kg) supplementation in sow diet increased the levels of IgG, IgA, and fat in sow milk and enhanced antioxidant and immune capacity in piglets with the upregulation of plasma IgG, IgA, T-AOC and CAT levels (94). Similarly, injection of 1000 IU vitamin E during gestation also increases serum IgG in sows (100).

Polyphenol is a bioactive substance with antioxidant, anticancer, anti-inflammatory and antibacterial properties (101). Supplementation of grape seed polyphenols (GSP) (300mg/kg) during late pregnancy and lactation reduced the number of dead fetuses, improved farrowing and pre-weaning survival (91). This might due to GSP increased antioxidant ability, progesterone and estradiol levels as well as the content of colostral IgM and IgG in sow (91). Intriguingly, effects of GSP on colostral immunoglobin production is better than vitamin E (91). Supplementation herbal extracts during pregnancy and lactation also enhance the immune function and antioxidant capacity of next generation through maternal-offspring transmission. *Forsythia suspensa* extract (FSE) is a medicinal herb extract that mainly consists of forsythiaside A, forythialan A, phillyrin and phillygenin. FSE has been shown to perform antioxidant (102), intestinal microflora-regulating, and anti-inflammatory effects (103). Dietary supplementation with FSE (100mg/kg) in sows from the G85 to farrowing could upregulate the milk fat, milk protein and IgM level in colostrum, and increase the immune ability of the piglets (104). Mechanistically, FSE limits the inflammatory response with the inhibition of NF-κ B signaling and the activation of Nrf2/HO-1 pathway (105). In addition, GE has an anti-inflammatory effect by inhibiting the expression of chemokines (106). The sow feed GE could improve the content of antioxidant and phenolic compounds in piglets’ plasma, and enhance the immune function by improve the concentration of IgG in colostrum and the plasma of the piglets (107). Resveratrol is a plant polyphenol with anti-inflammatory and antioxidant properties (108). Resveratrol (300 mg/kg) supplementation in sow diet improved the intestinal morphology and reduced intestinal inflammation as well as diarrhea in the offspring (109).

As an essential trace element for sows, selenium (Se) is incorporated into selenopsroteins and subsequently prevent intestinal inflammation by alleviating oxidative stress (110). In addition, selenoproteins such as glutathione peroxidase (GPX) and thioredoxin reductase (TXNRD) play an important role in the regulation of immune function (111). Organic Se compounds are more bioavailable than inorganic Se forms (112, 113). Supplementing sow gestation diets with HMSeBA (0.3 mg Se/kg) increases the expression of antioxidant-related selenoprotein genes in the placenta (*GPx2*, *GPx3*) and liver of neonates (*GPx1*, *GPx2*, *GPx3* and *TXNRD2*). Furthermore, administration of HMSeBA decreased the gene expression of *IL-1β*, *IL-6* and *IL-8* in placentas and IL-6 serum concentration in neonatal piglets. Therefore, HMSeBA supplementation in sows during late pregnancy increased the antioxidant capacity of piglets and reduced maternal and fetal inflammation (114). Similarly, another study reported that HMSeBA (0.3 mg Se/kg) supplementation to sows during pregnancy could up-regulate GPX1, GPX4 and selenoprotein expressions in the thymus and spleen of the offspring. Besides, the levels of inflammation, autophagy and endoplasmic reticulum stress were reduced, suggesting favorable outcomes in the immune function of offspring (115). Moreover, provision of maternal hydroxy-selenomethionine (OH-SeMet) (0.3 mg Se/kg) during G84 to L21 showed a significantly increase of IgG level in piglets at weaning (2).

Taurine (Tau), a metabolite of methionine and cysteine, have anti-inflammatory and antioxidant properties (116, 117). Tau effectively promotes mammalian growth and intestinal development (118). Supplementation with Tau (1%) in sow diets from G75 to weaning could significantly increase the activity of antioxidant enzymes (T-SOD, T-AOC, and CAT) in piglet serum and weaning body weight of the piglets. Besides, the height of jejunal villi, the ratio of villi height to crypt depth (VCR) and the expression of tight junction were also increased (95).

Oxidized β-carotene (OxBC) is a complex mixture produced by complete and spontaneous oxidation of β-carotene. The addition of OxBC (8 mg/kg) to the perinatal diet (G85-L21) improved the litter weight and individual body weight of the weaned piglets. This might be due to OxBC increased the immune status of sows, which further affect the growth of piglets. This is evidenced by decreased levels of cytokines (TNF-α and IL-18) and increased levels of immunoglobulin (IgM, IgA, and IgG) in colostrum (92).

## Other Nutritional Strategies

In this section, we describe some other nutrients which are advantageous to regulate the immunity and inflammation of piglets when supplemented in sow diets such as rare earth elements, lysozyme, and yeast nucleotides etc (**Table 3**).

Rare earth elements (REEs) includes 15 elements such as lanthanum (La) and cerium (Ce) (119). In addition to promote growth and feed conversion rate, rare earth elements also have anti-inflammatory and antioxidant properties (120, 121). A recent study showed that maternal supplementation with REEs (200 mg/kg) during late gestation could improve the antioxidant capacity and immune system through the up-regulation of serum CAT and GSH-Px level and downregulation of the serum TNF-α level of sow. In addition, piglets from REEs fed sow, have higher uniformity of birth weight and weaning weight, which might be related to the higher serum IGF-1 level (93). Furthermore, increased abundance of beneficial bacteria (*Christensenellaceae* and *Ruminocococaceae*) and decreased abundance of opportunistic pathogenic bacteria (*Proteus* and *Campylobacter*) were also found in the intestinal tract of piglets (93).

Lysozyme (LZM) is a natural antibacterial enzyme found in the tears, saliva and milk of mammals (122). Previous studies have shown that lysozyme has multiple beneficial effects on piglets, including improving intestinal morphology (123), regulating the intestinal microflora (124), and improving immunity (125). Sows fed diets containing lysozyme (300 g/t) from late gestation to weaning exhibited shorter weaning-estrous intervals and less stillbirths. In addition, serum IgM, IgA, IgG and IL-1 in sow were increased during lactation. Correspondingly, serum IgA, IgG, IgM, and IL-10 concentrations were also increased in piglet (96). Besides, piglets showed reduced rates of diarrhea, which may be due to a decreased number of *campylobacter* in the feces (126).

Nucleosides could promote the growth and development of intestinal epithelial cells (127). The addition of nucleotides to infant formula has a protective effect in preventing diarrhea and improving immunity (128). As a byproduct of yeast degradation, yeast-based nucleotides (YN) are rich in nucleotides. Supplementation of yeast cultures during pregnancy and lactation decrease of diarrhea and improve the growth performance of piglets (129). In detail,administration of yeast-based nucleotide (4 g YN/kg) during late pregnancy and lactation (G85-L20) of sow improved the development of intestinal morphology, and increased innate immunity with upregulation of intestinal IL-17, IL -8, IL -1β, IL -10 and TNF-α expressions in neonatal piglets (97).

Spray-dried plasma (SDP) is a protein-rich feed additive that contains immunoglobulins, peptides, glycoproteins and other active ingredients (130). Previous studies have shown that supplementation of SDP improved the immune response of pigs (131). From late pregnancy to weaning (G85-L27), maternal supplemented with 1% SDP reduced the serum concentrations of TNF-α, TGF-β1 and cortisol in sows and serum concentrations of TNF-α, TGF-β1 and cortisol in piglets. Additionally, the average daily gain of piglets at weaning was greater, and serum concentrations of cortisol, TGF-β1, TNF-α and C- reactive protein were lower (132).

## Conclusion and Outlook

Dietary fiber regulates inflammatory and immune response in the offspring by modulating the maternal intestinal microflora and milk immunoglobulin content. The antioxidant substances could directly react with the free radicals and enhance the maternal antioxidant capacity, thereby indirectly reducing infection in the offspring. The oil and fat products not only provide adequate energy to sows, but also supply functional fatty acids to alleviate infection and enhance the immune function in the offspring by exerting the anti-inflammatory and anti-oxidant effects. In summary, maternal nutrition intervention is an effective way to regulate the inflammatory response and immunity in the offspring.

In this review, we mainly focus on the positive effects of nutrients in the regulation of immunity and inflammatory response of sows and piglets during pregnancy and lactation. It worth noting that these effects would be affected by timing and/or dosage of nutrient supplementation. Moreover, it is well known that excessive addition of fat usually has a negative effect on pigs. The toxic effects of excessive addition of other products, such as vitamin E and selenium (133) are also worthy of attention. Therefore, we have given the current dosage of these products. However, the adverse effects of excessive maternal supplementation of such products on the immune system of piglets still need further research. In addition, applying nutrients to piglets and sows at the same time during lactation could produce better results (93). Even though nutrient mixture might produce synergistic and addictive effects, but economic cost should be considered in pig production. Future study needs to identify the best time and dosage for nutrient supplementation in sow diet. In addition, current studies only observe the change of phenotypic indicators, *in vitro* cell experiments are required to clarify the potential mechanism. Lastly, whether the metabolites of these nutrients were involved in the regulation of immunity and inflammation in the offspring is still unclear and require more research.

## Author Contributions

QL, SZ, and MR initiated the idea, the scope, and the outline of this review paper. QL, SY, XZ, XL, ZW, YQ, WG, MR, and SZ studied and analyzed all of the publications cited in this paper and were involved in the manuscript preparation. SZ and MR conducted the final editing and proofreading. All authors contributed to the article and approved the submitted version.

## Funding

This study was financially supported by the National Natural Science Foundation of the P.R. of China (No. 31872364 and No. 31802067), Guangdong Basic and Applied Basic Research Foundation (No. 2021A1515010440), Science and Technology Program of Guangzhou (No. 202102020056), Anhui Provincial Science and Technology Major Special Project (201903a06020002).

## Conflict of Interest

The authors declare that the research was conducted in the absence of any commercial or financial relationships that could be construed as a potential conflict of interest.

## Publisher’s Note

All claims expressed in this article are solely those of the authors and do not necessarily represent those of their affiliated organizations, or those of the publisher, the editors and the reviewers. Any product that may be evaluated in this article, or claim that may be made by its manufacturer, is not guaranteed or endorsed by the publisher.
